# Elevated urinary succinate in women with symptom-defined overactive bladder without clinically recognized cardiometabolic disease: a cross-sectional study

**DOI:** 10.1186/s12905-026-04596-8

**Published:** 2026-06-26

**Authors:** Ömer Faruk Öz, Mehmet Murat Seval, Özlem Doğan, Bulut Varli, Şerife Esra Çetinkaya, Fulya Dökmeci

**Affiliations:** 1 Department of Obstetrics and Gynecology, Akdeniz University School of Medicine, Antalya, Turkey; 2 Department of Obstetrics and Gynecology, Ankara University School of Medicine, Mamak, Ankara, Turkey; 3 Ankara University Graduate School of Health Sciences, Ankara, Turkey; 4 Department of Medical Biochemistry, Ankara University School of Medicine, Ankara, Turkey

**Keywords:** Overactive bladder, Urinary metabolites, Succinate, Malate, Tricarboxylic acid cycle, Biomarkers

## Abstract

**Background:**

Overactive bladder (OAB) is a symptom-defined syndrome, and its metabolic correlates remain incompletely understood. This study investigated whether urinary tricarboxylic acid cycle metabolites differ between women with symptom-defined probable OAB and controls without clinically recognized cardiometabolic disease.

**Methods:**

In this cross-sectional clinic-based study, women aged 18 years and older attending a gynecology outpatient clinic were enrolled. Participants with self-reported cardiometabolic disease or related medication use were excluded. Probable OAB was defined by an Overactive Bladder Awareness Tool Version 8 score of 8 or higher. Midstream urine samples were analyzed for succinate and malate by gas chromatography-mass spectrometry and normalized to urinary creatinine. Group comparisons, Spearman correlations, multivariable log-linear regression, sensitivity analyses, and exploratory receiver operating characteristic analyses were performed.

**Results:**

A total of 186 women were included, comprising 81 with OAB and 105 controls. Women with OAB were older, had slightly higher body mass index, higher parity, and were more frequently postmenopausal. Urinary succinate and malate levels were higher in the OAB group. After adjustment for age, body mass index, parity, and menopausal status, OAB remained associated with higher urinary succinate (adjusted geometric mean ratio, 1.69; 95% CI, 1.34–2.12) and malate (1.25; 95% CI, 1.02–1.52). Succinate showed modest exploratory discrimination (AUC, 0.678), whereas malate showed limited discrimination (AUC, 0.560).

**Conclusions:**

Urinary succinate was higher in women with symptom-defined probable OAB even after adjustment for major demographic covariates, while the association with malate was weaker. These findings are hypothesis-generating and require confirmation in adequately powered, prospectively phenotyped cohorts with standardized metabolic, dietary, and urinary microbiome assessment.

**Supplementary Information:**

The online version contains supplementary material available at https://doi.org/10.1186/s12905-026-04596-8.

## Background

Overactive bladder (OAB) syndrome is defined as urinary urgency, usually accompanied by increased daytime frequency and nocturia, with or without urgency urinary incontinence, in the absence of urinary tract infection or other identifiable pathology [1]. OAB is common: large epidemiological surveys and recent meta-analyses suggest a global prevalence of approximately 20% overall and 21.9% among women, with prevalence increasing with age and over time [2–4]. OAB is associated with substantial impairment in quality of life, adversely affecting sleep quality, emotional well-being, and mental health [5].

OAB is, by definition, a symptom-defined syndrome. A clinical diagnosis is therefore based on history, symptom questionnaires, and bladder diaries, supported by evaluation to exclude infection and other relevant pathology. Biomarkers are not required to establish the diagnosis; however, objective measures may help identify biological sub-phenotypes, refine risk stratification, and support mechanistic research and treatment monitoring [6, 7].

Increasing evidence implicates metabolic and inflammatory pathways in OAB pathophysiology. Biomarkers reflecting mitochondrial dysfunction, oxidative stress, inflammation, and neurotrophic signaling have been investigated in urine and blood; however, none have been adopted into routine clinical practice [8–12].

Epidemiological studies have reported a higher prevalence of OAB in individuals with cardiometabolic disorders, suggesting an association between metabolic dysregulation and bladder dysfunction [13]. Impaired energy metabolism and chronic low-grade inflammation, characteristic of metabolic disturbances, are associated with altered tricarboxylic acid (TCA) cycle activity and accumulation of intermediary metabolites [14]. In line with this concept, population-based analyses have demonstrated an inverse association between cardiovascular health, assessed by the Life’s Essential 8 score, and OAB prevalence [15, 16].

Among TCA cycle intermediates, succinate and malate have gained attention as biologically active metabolites that may accumulate under conditions of metabolic stress, mitochondrial dysfunction, and hypoxia. Malate has been associated with OAB symptom severity in metabolomic studies [8]. Succinate can also act as a signaling metabolite. Experimental work suggests that succinate can activate succinate receptor 1 (SUCNR1; previously GPR91) on urothelial cells and may modulate inflammatory and bladder signaling pathways in settings of metabolic stress [17–21].

In this context, succinate and malate, beyond their metabolic roles, represent plausible candidates for exploring metabolic correlates of OAB symptoms. However, most prior studies evaluating these metabolites in OAB have focused on individuals with overt metabolic syndrome or experimental models [18, 20, 21], limiting generalizability to women without clinically recognized cardiometabolic disease. OAB frequently occurs in women without diagnosed metabolic disorders, raising the possibility that subclinical metabolic alterations may be present and may not be adequately captured by body mass index (BMI).

This study therefore aimed to investigate urinary succinate and malate levels in women with and without OAB symptoms, excluding women with clinically recognized cardiometabolic disease, and to evaluate their associations with symptom severity and exploratory diagnostic performance. The female-only design reflected the gynecology outpatient setting and was intended to reduce sex-related heterogeneity in this exploratory urinary biomarker study.

## Methods

### Study design and participants

This cross-sectional study included women aged ≥ 18 years attending the gynecology outpatient clinic of Ankara University School of Medicine between March and August 2021, either for urinary/gynecological symptoms or for routine gynecological check-ups. During the study period, 442 women were screened for eligibility; some eligible women declined participation (e.g., unwillingness to complete the questionnaires and/or provide a urine sample). A total of 186 women were enrolled and included in the final analysis. Because recruitment was conducted in a gynecology clinic and because lower urinary tract symptoms, urinary metabolite profiles, and potential microbiome-related mechanisms may be influenced by sex-specific reproductive and menopausal factors, the study was intentionally limited to women.

Women were excluded if they were pregnant or had hematuria on routine urinalysis, urinary tract infection, urolithiasis, neurological or psychiatric disorders, cognitive impairment, liver or renal failure, malignancy, prior pelvic radiotherapy, pelvic organ prolapse stage ≥ 2, postvoid residual urine volume > 100 mL, or use of anticholinergic medications within the preceding three months. The medication exclusion was applied to minimize treatment-related effects on urinary symptoms and metabolite levels. Study participation did not delay or replace routine clinical care; after questionnaires and urine sampling, participants were managed according to standard clinic practice as clinically indicated.

Metabolic syndrome was not defined using formal consensus criteria and laboratory parameters were not systematically obtained. To minimize confounding from clinically recognized cardiometabolic disease, a practical clinical exclusion strategy was applied. Women were excluded if they reported a physician-diagnosed history of diabetes mellitus, hypertension, dyslipidemia, or impaired glucose tolerance, or if they were using antidiabetic, antihypertensive, or lipid-lowering medications.

After exclusion of ineligible participants, eligible women were informed about the study and written informed consent was obtained prior to enrollment. All methods were carried out in accordance with the relevant guidelines and regulations. The study protocol was approved by the Ankara University School of Medicine Ethics Committee (No: 13-208-21).

### Clinical assessment

Participants completed validated Turkish versions of the Overactive Bladder Awareness Tool Version 8 (OAB-V8) [22, 23], Urinary Distress Inventory-6 (UDI-6) [24, 25], Incontinence Impact Questionnaire-7 (IIQ-7) [24, 25], and Incontinence Severity Index (ISI) [26, 27]. Clinical examination findings and bladder diary parameters were collected in accordance with International Urogynecological Association recommendations [1]. The available diary dataset included daily fluid intake, daily micturition episodes, and daily incontinence episodes; however, urgency episodes were not recorded in a standardized manner sufficient to support a diary-based reclassification analysis. Body mass index was calculated for all participants but was not used as a metabolic exclusion criterion. Demographic variables, gravidity (number of pregnancies), parity (number of births; parity = 0 indicates nulliparity), and menopausal status were recorded at enrollment.

Women with an OAB-V8 score ≥ 8 were classified as the OAB group, while those with scores ≤ 7 comprised the control group. Because OAB-V8 is primarily an awareness/screening questionnaire, this classification should be interpreted as symptom-defined probable OAB rather than a definitive clinical diagnosis. Three-day bladder diary variables were used to characterize voiding frequency, fluid intake, and incontinence burden, but diary-based urgency criteria were not used as an independent diagnostic definition in the primary analysis.

### Urinary metabolite analysis

Midstream spot urine samples were obtained during the clinic visit after a bladder filling period of approximately 3–4 h, while avoiding excessive fluid intake. No standardized fasting period, dietary protocol, caffeine restriction, or recent physical-activity restriction was applied before sampling. Samples were centrifuged and stored at -80 °C until analysis. Urinary succinate and malate concentrations were measured using gas chromatography-mass spectrometry (GC-MS; Thermo Scientific, San Jose, CA, USA) and normalized to urinary creatinine (µmol/mmol creatinine).

### Statistical analysis

Statistical analyses were performed using the Statistical Package for the Social Sciences (SPSS) version 16. Analyses were exploratory. No formal a priori sample size calculation was performed; the sample size was determined by the number of eligible participants recruited during the predefined study period. With 81 women with OAB and 105 controls, the available sample provided approximately 80% power at a two-sided alpha level of 0.05 to detect a between-group standardized mean difference of about 0.41 in a simple two-sample comparison; therefore, smaller effects, subgroup analyses, and ROC-derived cut-offs should be interpreted cautiously. Group comparisons were conducted using Student’s t test or Mann-Whitney U test, as appropriate. Categorical variables were compared using chi-square or Fisher’s exact tests. Associations were assessed using Spearman correlation coefficients. Because urinary succinate and malate distributions were right-skewed, metabolite values were natural log-transformed for multivariable modeling. Multivariable linear regression models evaluated associations between OAB status and urinary metabolites while adjusting for age, BMI, parity, and menopausal status. Model estimates were back-transformed and reported as geometric mean ratios with 95% confidence intervals. Sensitivity analyses were performed in premenopausal women and in participants with BMI < 30 kg/m². Receiver operating characteristic (ROC) analysis was used only as an exploratory assessment of discrimination. A two-sided *p* value < 0.05 was considered statistically significant.

## Results

During the study period, 442 women were screened for eligibility. After exclusion of women who did not meet inclusion criteria, 186 participants provided written informed consent and were included in the final analysis. Based on OAB-V8 scores, 81 women were classified as having OAB and 105 women constituted the control group.

Women in the OAB group were older than controls (43 ± 11 vs. 35 ± 10 years, *p* < 0.001) and had higher BMI values (26.5 ± 2.4 vs. 25.5 ± 2.7 kg/m², *p* = 0.010). The OAB group also had higher parity (median 2 vs. 0, *p* < 0.001) and a higher proportion of postmenopausal women (26% vs. 10%, *p* = 0.006) (Table 1).

**Table 1:** Baseline characteristics by OAB status

	Control (*n* = 105)	OAB (*n* = 81)	*p*
Age (years)﻿			
*Mean ± SD*	35 ± 10	43 ± 11	< 0.001^a^
*Median (range)*	33 (21–66)	43 (21–73)	
Body mass index (kg/m^2^)			
*Mean ± SD*	25.5 ± 2.7	26.5 ± 2.4	0.010^a^
*Median (range)*	25 (18–32)	26 (21–32)	
Gravidity (number of pregnancies)			
*Mean ± SD*	1.4 ± 1.4	2.5 ± 1.4	< 0.001^a^
*Median (range)*	1 (0–5)	2 (0–6)	
Parity (number of births)			
*Mean ± SD*	0.9 ± 1.2	2.1 ± 1.2	< 0.001^b^
*Median (range)*	0 (0–4)	2 (0–5)	
Post-menopausal women			
*n (%)*	11 (10)	21 (26)	0.006^c^

Data are presented as mean ± SD and median (range) for continuous variables, and as n (%) for categorical variables. Parity includes 0 (nulliparous). *P* values were calculated using Student’s t testᵃ, Mann–Whitney U testᵇ, or chi-square testᶜ, as appropriate

*OAB* overactive bladder, *BMI* body mass index, *SD* standard deviation

The scores from the validated questionnaires are summarized in Table 2. Median OAB-V8, UDI-6 (total and subscales), IIQ-7, and incontinence severity index scores were all markedly elevated in the OAB group (*p* < 0.001 for all). According to the three-day bladder diary, daily fluid intake did not differ significantly between groups (*p* = 0.340). However, the OAB group reported significantly more daily micturition episodes (9.7 ± 1.8 vs. 7.2 ± 1.6, *p* < 0.001) and daily incontinence episodes (1.7 ± 1.2 vs. 0.4 ± 0.6, *p* < 0.001) compared with the control group (Table 2).

**Table 2:** Clinical findings by OAB status

	Control (*n* = 105)	OAB (*n* = 81)	*p*
Questionnaires			
Overactive Bladder Awareness Tool Version 8			
*- Total score*,* Median (range)*	6 (1–7)	20 (8–35)	< 0.001 ^b^
Urinary Distress Inventory-6			
*- Total score*,* Median (range)*	17 (0–42)	64 (33–96)	< 0.001 ^b^
*- Irritative subscale*,* Median (range)*	25 (0–63)	75 (38–100)	< 0.001 ^b^
*- Stress subscale*,* Median (range)*	12 (0–50)	75 (25–100)	< 0.001 ^b^
*- Obstructive subscale*,* Median (range)*	12 (0–63)	50 (0-100)	< 0.001 ^b^
Incontinence Impact Questionnaire-7			
*- Total score*,* Median (range)*	9 (0–29)	67 (29–100)	< 0.001 ^b^
Incontinence severity index			
*- Total score*,* Median (range)*	2 (1–6)	9 (4–12)	< 0.001 ^b^
Three-day bladder diary variables			
Daily fluid intake (L)			
*Mean ± SD*	3.1 ± 0.6	3.2 ± 0.6	0.340 ^a^
*Median (range)*	3.0 (1.9–4.4)	3.2 (1.9–4.5)	
Daily micturition episodes			
*Mean ± SD*	7.2 ± 1.6	9.7 ± 1.8	< 0.001 ^a^
*Median (range)*	7 (3–11)	9 (7–15)	
Daily incontinence episodes			
*Mean ± SD*	0.4 ± 0.6	1.7 ± 1.2	< 0.001 ^a^
*Median (range)*	0 (0–2)	1 (0–4)	

Data are presented as median (range) for questionnaire scores and as mean ± SD and median (range) for bladder diary variables. *P* values were calculated using Student’s t testᵃ or Mann–Whitney U testᵇ, as appropriate

*OAB* overactive bladder, *L* Liter, *SD* standard deviation

Results from the mass spectrometric analysis, summarized in Table 3, indicate that the mean urinary succinate concentration was significantly higher in individuals in the OAB group (4.33 ± 3.46 µmol/mmol creatinine) than in the control group (3.09 ± 3.24 µmol/mmol creatinine; *p* = 0.013). Similarly, urinary malate levels were elevated in the OAB cohort (0.73 ± 0.53 µmol/mmol creatinine) relative to controls (0.58 ± 0.32 µmol/mmol creatinine; *p* = 0.035).

**Table 3:** Urinary metabolite levels by OAB status

	Control (*n* = 105)	OAB (*n* = 81)	*p*
Urinary succinate (µmol/mmol creatinine)			
*Mean ± SD*	3.09 ± 3.24	4.33 ± 3.46	0.013 ^a^
*Median (range)*	2.17 (0.49–23.58)	3.25 (0.96–22.05)	
Urinary malate (µmol/mmol creatinine)			
*Mean ± SD*	0.58 ± 0.32	0.73 ± 0.53	0.035 ^a^
*Median (range)*	0.49 (0.06–1.66)	0.58 (0.11–2.60)	

Data are presented as mean ± SD and median (range). Metabolite concentrations were normalized to urinary creatinine and are reported as µmol/mmol creatinine. *P* values were calculated using Student’s t testᵃ

*OAB* overactive bladder, *SD* standard deviation

In the correlation analysis, urinary succinate levels demonstrated a significant weak positive correlation with OAB-V8, IIQ-7, and incontinence severity index scores, as well as with daily fluid intake, daily micturition, and incontinence episodes recorded in the three-day bladder diary (Table 4). Urinary succinate levels also showed a significant moderate positive correlation with the total and irritative subscale scores of the UDI-6. Similarly, urinary malate levels exhibited a significant weak positive correlation with OAB-V8, UDI-6 total and irritative subscale scores, incontinence severity index, and daily fluid intake, micturition, and incontinence episodes in the bladder diary (Table 4).

**Table 4:** Correlations of urinary metabolites with clinical variables

	Urinary succinate level		Urinary malate level	
	Correlation Coefficient	*p*	Correlation Coefficient	*p*
Age	-0.025	0.740	-0.111	0.131
Body-mass index	0.003	0.973	-0.013	0.864
Gravidity (number of pregnancies)	0.040	0.584	-0.104	0.160
Parity (number of births)	0.051	0.493	-0.081	0.271
OAB-V8 score	0.380	< 0.001	0.157	0.032
UDI-6 total score	0.455	< 0.001	0.182	0.013
UDI-6 irritative subscale	0.419	< 0.001	0.215	0.003
UDI-6 stress subscale	0.301	< 0.001	0.114	0.121
UDI-6 obstructive subscale	0.342	< 0.001	0.051	0.485
IIQ-7 score	0.373	< 0.001	0.128	0.081
Incontinence severity index	0.375	< 0.001	0.178	0.015
Daily fluid intake (L)	0.325	< 0.001	0.240	0.001
Daily micturition episodes	0.343	< 0.001	0.160	0.029
Daily incontinence episodes	0.375	< 0.001	0.224	0.005

Values are Spearman correlation coefficients (ρ) with corresponding *p* values. OAB, overactive bladder; OAB-V8, Overactive Bladder Awareness Tool Version 8; UDI-6, Urinary Distress Inventory-6; IIQ-7, Incontinence Impact Questionnaire-7

Because metabolite distributions were right-skewed, log transformation was applied for multivariable modeling. In multivariable linear regression adjusting for age, BMI, parity, and menopausal status, OAB status remained associated with higher urinary succinate (geometric mean ratio 1.69, 95% CI 1.34–2.12; *p* < 0.001) and malate (ratio 1.25, 95% CI 1.02–1.52; *p* = 0.030) (Table 5). In sensitivity analyses restricted to premenopausal women and to participants with BMI < 30 kg/m², the association with succinate remained statistically significant, whereas the malate association was weaker and did not consistently reach statistical significance (Supplementary Table S1).

**Table 5:** Multivariable associations between OAB and urinary metabolites

Urinary Metabolite	Adjusted ratio (OAB vs. control)	95% CI	*p* value
Urinary succinate	1.69	1.34–2.12	< 0.001
Urinary malate	1.25	1.02–1.52	0.03

Values are presented as adjusted geometric mean ratios (OAB vs. control) with 95% confidence intervals (CI) from multivariable linear regression models of log-transformed metabolite levels. Models were adjusted for age, body mass index (BMI), parity, and menopausal status

*OAB* overactive bladder, *TCA* tricarboxylic acid

**Fig. 1:**
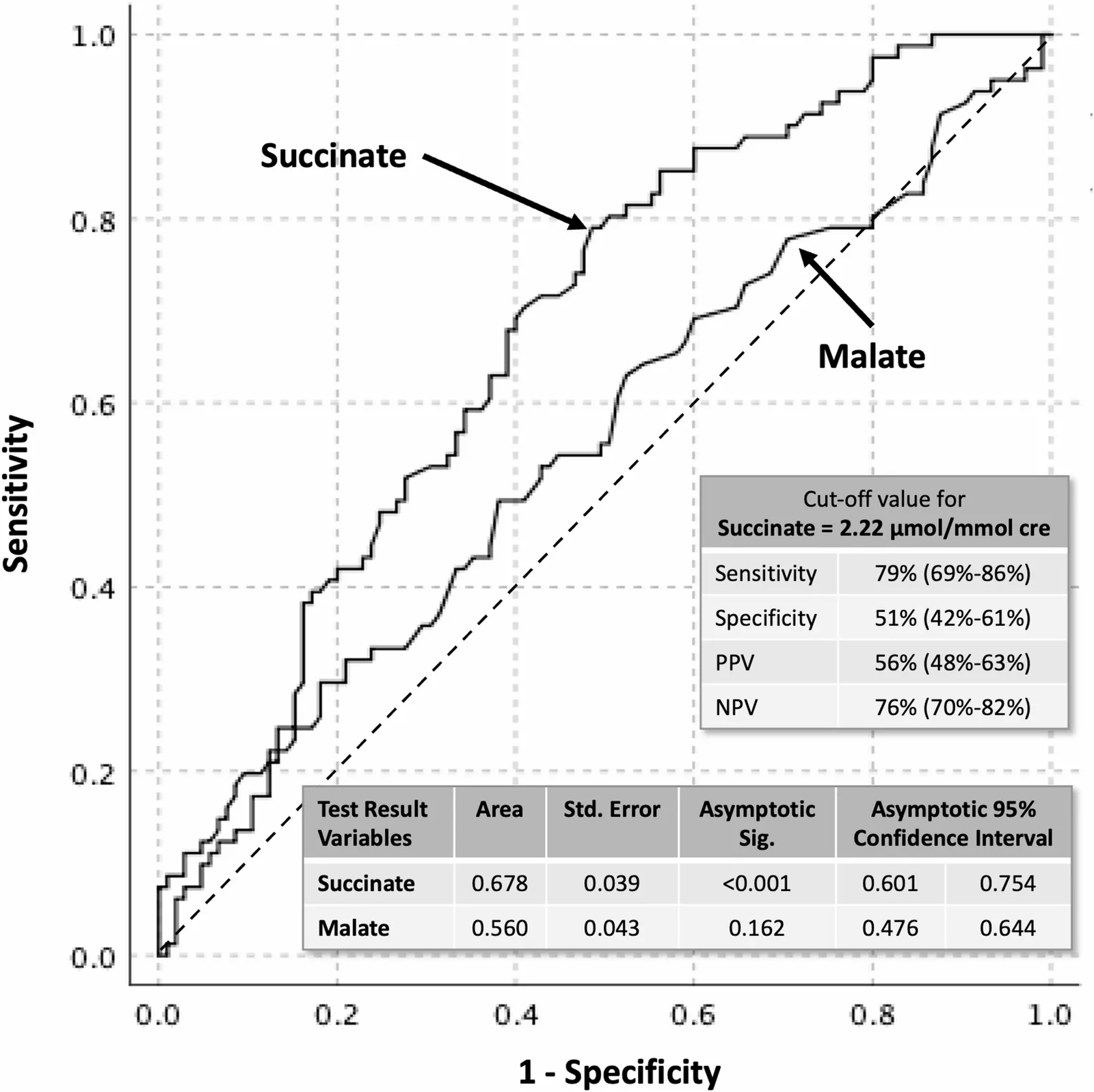
Exploratory ROC curves for urinary succinate and malate. Receiver operating characteristic (ROC) curves for urinary succinate (primary) and malate (secondary) to discriminate women with symptom-defined overactive bladder (OAB) from controls without clinically recognized cardiometabolic disease. The area under the curve (AUC) for succinate was 0.678 (95% confidence interval [CI], 0.601–0.754). The Youden index-based cut-off value was 2.22 µmol/mmol creatinine, yielding a sensitivity of 79% (95% CI, 69–86%), specificity of 51% (42–61%), positive predictive value (PPV) of 56% (48–63%), and negative predictive value (NPV) of 76% (70–82%). The AUC for malate was 0.560 (95% CI, 0.476–0.644). These results indicate modest discriminatory performance for succinate and limited discrimination for malate in this exploratory cohort

Exploratory ROC curves of succinate and malate values for discriminating women with symptom-defined OAB from controls are shown in Fig. 1. For succinate, the cut-off value was 2.22 µmol/mmol creatinine (AUC 0.678 [0.601–0.754]; sensitivity 79%, specificity 51%, PPV 56% and NPV 76%). Urinary malate showed limited discrimination (AUC 0.560 [95% CI 0.476–0.644]) (Fig. 1).

## Discussion

In this cross-sectional clinic-based cohort of women without clinically recognized cardiometabolic disease, urinary succinate and, to a lesser extent, malate were higher in women with symptom-defined probable OAB than in controls. The association between OAB status and urinary succinate persisted after adjustment for age, BMI, parity, and menopausal status and was broadly consistent in sensitivity analyses. In contrast, the malate association was smaller and less stable, suggesting that malate should be interpreted as a secondary and more exploratory finding.

These findings add to the growing literature suggesting that OAB symptoms may be linked to metabolic and inflammatory pathways. Succinate is not only a TCA-cycle intermediate but also a signaling metabolite that can accumulate during metabolic stress, hypoxia, or mitochondrial dysfunction and can activate SUCNR1/GPR91-mediated pathways. Experimental studies have reported SUCNR1 expression and succinate-related signaling in urothelial and bladder smooth-muscle models, including effects on inflammatory signaling, beta-3 adrenergic pathways, and detrusor relaxation [17–21]. Our results do not establish causality, but they support the hypothesis that urinary succinate may reflect a metabolic or inflammatory correlate of OAB symptom burden.

In our cohort, the weaker and less consistent findings for malate suggest that it may represent a less specific metabolic signal than succinate. Malate participates in mitochondrial energy metabolism and can vary with broader systemic and urinary metabolic conditions. Although malate was higher in unadjusted comparisons and remained modestly associated with OAB in the main adjusted model, its correlations with symptom scores were weaker and sensitivity analyses were less consistent. This pattern suggests that succinate may be the more biologically informative candidate for future studies.

The phrase “without clinically recognized cardiometabolic disease” is important. Participants were excluded based on known physician-diagnosed cardiometabolic disease and related medication use rather than by systematic laboratory screening. Therefore, undiagnosed insulin resistance, dyslipidemia, hypertension, central adiposity, or other subclinical metabolic abnormalities may have been present. BMI was slightly higher in the OAB group and was adjusted for in the primary model, but BMI is an imperfect surrogate for metabolically relevant adiposity. Future studies should incorporate waist circumference, blood pressure, fasting glucose or HbA1c, lipid profile, inflammatory markers, and more detailed medication history [28, 29].

The symptom definition of OAB is another key consideration. In this study, OAB grouping was based on the validated Turkish OAB-V8 screening threshold. Diary variables showed that the OAB group had substantially higher micturition and incontinence frequency, supporting a clinically meaningful symptom-burden contrast. However, OAB-V8 is an awareness and screening instrument rather than a definitive diagnostic tool. Because urgency episodes were not recorded in a standardized way in the available diary dataset, a reliable post hoc reclassification using a combined diary-based definition (for example, ≥ 8 voids/day with urgency) could not be performed. Future work should combine standardized clinical evaluation, bladder diaries that capture urgency episodes, and validated symptom questionnaires to reduce misclassification.

The absolute metabolite concentrations also require cautious interpretation. Succinate and malate values in both groups were not suggestive of marked pathological accumulation and should be interpreted in the context of previously reported urinary organic-acid variability and reference-value studies [30, 31]. Therefore, the between-group difference does not necessarily imply pathological metabolite accumulation. Rather than a stand-alone diagnostic abnormality, the observed shift may represent a modest metabolic signal associated with OAB symptoms. This is consistent with the modest ROC performance of succinate and the limited discrimination of malate.

Specimen collection may have influenced urinary metabolite levels. Creatinine normalization was used to reduce the effect of urine concentration, and daily fluid intake did not differ significantly between groups. Nevertheless, spot urine metabolites can be affected by fasting state, diet, caffeine intake, physical activity, circadian timing, muscle mass, and hydration [32]. Because standardized dietary, fasting, and recent physical-activity restrictions were not applied, these factors should be considered potential sources of residual variability and should be controlled in future prospective studies.

The urinary microbiome provides an additional plausible mechanism. Studies using enhanced culture and sequencing methods have shown that the female bladder may harbor polymicrobial communities, and differences in urinary microbiome composition and diversity have been associated with urgency urinary incontinence and symptom severity [33–35]. Succinate can also be influenced by host-microbe metabolic interactions [36]. Because this study did not include urinary microbiome profiling, we cannot determine whether the higher urinary succinate observed in women with OAB reflects host cellular metabolism, microbial metabolism, or both. Integrated metabolomic-microbiome studies are needed to examine this possibility.

The clinical implications are therefore hypothesis-generating. Succinate showed only modest exploratory discrimination and limited specificity, indicating that it should not be interpreted as a diagnostic biomarker for OAB in its current form. Its potential value may instead lie in helping to identify metabolic or inflammatory sub-phenotypes of OAB when combined with clinical, anthropometric, laboratory, and microbiome data.

This study has several limitations. First, the sample size was modest and no formal a priori power calculation was performed, limiting precision for smaller effects, subgroup analyses, and diagnostic-threshold estimation. Second, the cross-sectional design precludes causal inference. Third, the groups differed in age, BMI, parity, and menopausal status, and residual confounding may persist despite multivariable adjustment. Fourth, cardiometabolic disease was excluded using clinical history and medication use rather than standardized metabolic phenotyping, so subclinical cardiometabolic abnormalities cannot be excluded. Fifth, OAB was defined using a screening questionnaire rather than a combined clinical and diary-based definition, and urgency episodes were not available for diary-based reclassification. Sixth, spot urine sampling was not standardized for fasting, diet, caffeine, circadian timing, or recent physical activity, and urine osmolality or specific gravity was not available for alternative normalization. Finally, urinary microbiome data were not collected. These limitations should be addressed in adequately powered prospective studies with standardized phenotyping and external validation.

## Conclusions

Urinary succinate was higher in women with symptom-defined probable OAB and remained independently associated after adjustment for age, BMI, parity, and menopausal status in this cohort without clinically recognized cardiometabolic disease. The association with malate was weaker. These findings support a hypothesis-generating link between OAB symptoms and urinary metabolic alterations, but they require confirmation in larger prospective cohorts with standardized cardiometabolic, dietary, bladder diary, and urinary microbiome assessment.

## Supplementary Information

Supplementary Material 1.

## Data Availability

The datasets used and/or analyzed during the current study are not publicly available due to privacy and ethical restrictions but are available from the corresponding author on reasonable request.

## Abbreviations

BMI: Body mass index
CI: Confidence interval
GC-MS: Gas chromatography-mass spectrometry
IIQ-7: Incontinence Impact Questionnaire-7
ISI: Incontinence Severity Index
OAB: Overactive bladder
OAB-V8: Overactive Bladder Awareness Tool Version 8
ROC: Receiver operating characteristic
SPSS: Statistical Package for the Social Sciences
SUCNR1: Succinate receptor 1
TCA: Tricarboxylic acid
UDI-6: Urinary Distress Inventory-6
