# Supplementary material for: Elevated urinary succinate in women with symptom-defined overactive bladder without clinically recognized cardiometabolic disease: a cross-sectional study
Source: BMC Womens Health. 2026 Jun 26;26:450. doi: 10.1186/s12905-026-04596-8 (PMC13563909; doi:10.1186/s12905-026-04596-8)
Supplement: Supplementary file 1 [file 12905_2026_4596_MOESM1_ESM.docx]

**Supplementary Table S1.** Sensitivity analyses of OAB-metabolite associations.

| Subgroup | Outcome | Adjusted ratio (OAB vs control) | 95% CI | p value |
| --- | --- | --- | --- | --- |
| Premenopausal (MNP = 0) | Urinary succinate | 1.56 | 1.20–2.02 | < 0.001 |
| Premenopausal (MNP = 0) | Urinary malate | 1.20 | 0.97–1.49 | 0.097 |
| BMI < 30 kg/m² | Urinary succinate | 1.63 | 1.29–2.07 | < 0.001 |
| BMI < 30 kg/m² | Urinary malate | 1.22 | 1.00–1.50 | 0.055 |

Values are presented as adjusted geometric mean ratios (OAB vs control) with 95% confidence intervals (CI) from multivariable linear regression models of log-transformed metabolite levels. Premenopausal models were adjusted for age, BMI, and parity. BMI < 30 kg/m² models were adjusted for age, BMI, parity, and menopausal status.
